# Supplementary material for: Pre-Transplant Alpha-Fetoprotein > 25.5 and Its Dynamic on Waitlist Are Predictors of HCC Recurrence after Liver Transplantation for Patients Meeting Milan Criteria
Source: Cancers (Basel). 2021 Nov 27;13(23):5976. doi: 10.3390/cancers13235976 (PMC8656660; doi:10.3390/cancers13235976)
Supplement: Supplementary file 1 [file cancers-13-05976-s001.zip › cancers-1464737-supplementary.pdf]

cancers

MDPI

Article

Pre-Transplant Alpha-Fetoprotein > 25.5 and Its Dynamic on Waitlist Are Predictors of HCC Recurrence after Liver Transplantation for Patients Meeting Milan Criteria

Bianca Magro, Domenico Pinelli, Massimo De Giorgio, Maria Grazia Lucà, Arianna Ghirardi, Alessandra Carrobio, Giuseppe Baronio, Luca Del Prete, Franck Nounamo, Andrea Gianatti, Michele Colledan and Stefano Fagiuoli

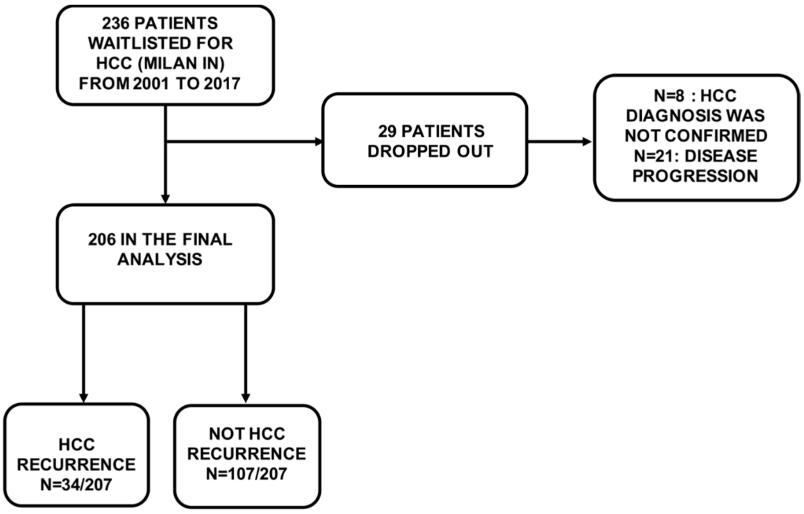

Figure S1. Patient selection.

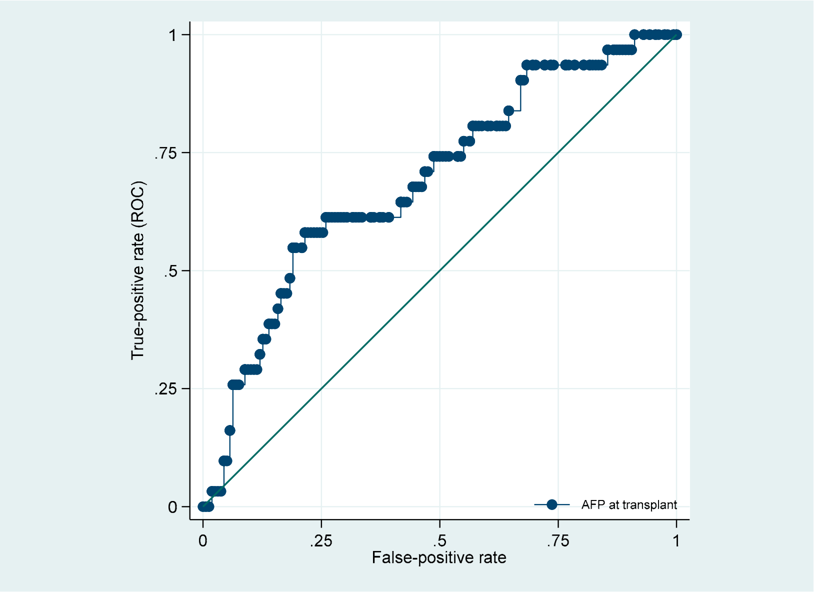

Figure S2. Receiving operating characteristics curve of AFP at transplant for the discrimination of cases of recurrences AUC = 0.69, 95% CI: 0.58–0.80.

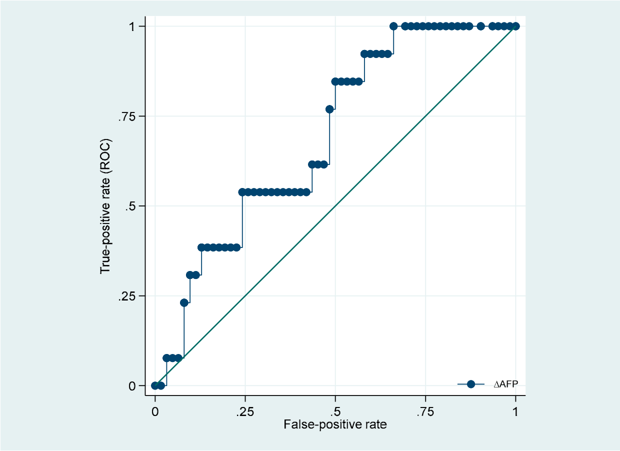

Figure S3. Receiving operating characteristics curve of relative increase in AFP values from entry to transplant (ΔAFP) for the discrimination of cases of recurrences (AUC = 0.69, 95% CI 0.55–0.83).

Table S1. All causes of mortality.

Causes.	N	DEATH TOT (*N* = 75)
HCC	22	29,3%
NOT HCC	53	70,7%
PNF	*2*	*2,7%*
Infections	*11*	*14,7%*
Surgical	*1*	*1,3%*
Cardiovascular	*12*	*16,0%*
Neoplasia	*12*	*16,0%*
Cirrhosis recurrence	*9*	*12,0%*
Others	*6*	*8,0%*
